# Is routine intravenous access commonly established before interventional pain procedures? Results of a spine intervention society practice pattern survey

**DOI:** 10.1016/j.inpm.2023.100246

**Published:** 2023-04-05

**Authors:** Margaret Beckwith, Zachary L. McCormick, Andrew Joyce, Masaru Teramoto, Shanalee Mountan, Daniel M. Cushman, Taylor Burnham, Aaron Conger

**Affiliations:** a Department of Physical Medicine and Rehabilitation, University of Utah School of Medicine, Salt Lake City, UT, USA; b Providence Physiatry Clinic, Portland, OR, USA

**Keywords:** Interventional pain, Procedures, Sedation, Intravenous access

## Abstract

**Summary of background data:**

Establishing routine intravenous (IV) access prior to interventional pain procedures performed without sedation is controversial. Anecdotally, practices very in terms of their use of routine IV access.

**Objectives:**

To determine the frequency with which routine IV access is obtained for various common interventional pain procedures among interventional pain providers.

**Methods:**

An anonymous survey was distributed to physician members of the Spine Intervention Society (SIS) on 08/13/2020, and remained open until 10/13/2020. Respondents provided information regarding demographics and current practice patterns.

**Results:**

141 SIS members completed the survey. A bimodal distribution was noted for most procedures, with most providers obtaining routine IV access either 0% of the time or 81–100% of the time. Routine IV access was used more frequently when performing cervical spine procedures, splanchnic or hypogastric plexus blocks, lumbar sympathetic blocks, and spinal cord stimulator trials. Excluding cases where IV's were used for sedation, the only procedures which providers obtained routine IV access more often than not were spinal cord stimulator trials, splanchnic/celiac plexus blocks, and hypogastric plexus blocks. Over half (52.5%) of respondents reported using an IV for an emergent situation in their career. While most providers would not modify their IV usage based on practice location, those that would were significantly more likely to decrease IV use if working in clinic-based procedures suites (*n* ​= ​25) compared to hospital-based surgical centers (*n* ​= ​2) or ambulatory surgical centers (*n* ​= ​9, p ​< ​0.001).

**Discussions/conclusions:**

There remains wide variation among practitioners in establishing routine intravenous access prior to interventional procedures. Most providers surveyed do not routinely establish IV access for the majority of interventional pain procedures. Routine IV access is more commonly obtained for cervical spine procedures than other spinal regions. Overall, complications precipitating the need for IV use are rare in interventional spine but likely to happen over the course of a career.

## 1 Introduction

The utilization of interventional procedures for treating various pain conditions has increased rapidly over the past two decades [[1], [2], [3]]. Despite this increase in utilization, there remains no consensus on whether establishing intravenous (IV) access is necessary for various interventional procedures when IV sedation is not used. Additionally, no study or survey has reported how frequently interventionalists utilize an IV during emergency situations.

Rare but significant risks of interventional spine procedures include seizure, anaphylaxis, spinal block, and/or arrhythmia, which may require rapid administration of medications or fluids intravenously. Establishing IV access prior to performance of interventional pain procedures may result in better outcomes should a serious medical event occur that would require urgent administration of medications or fluids (seizure, anaphylaxis, severe hypotension, or arrythmia). However, evidence-based benefits of routinely obtaining IV access this setting have not been established [4]. When evaluating the necessity of routine, pre-procedure IV access, the cumulative risks, costs, and patient discomfort of obtaining IV access in all patients who undergo interventional procedures must also be considered. Intravenous cannulation can be painful, and studies have shown that patients experience increased pain in association with multiple IV attempts [5]. This pain may increase anxiety and discomfort prior to the interventional procedure [5]. Intravenous access may result in infection, thrombophlebitis, infiltration injury, hematoma, and vascular injury [6]. Establishing IVs routinely leads to increased costs associated with lost staff time, lost procedure time, and supplies may range from $5 to $20 per IV [4,6].

Anecdotally, there appears to be wide variation among interventionalists; while some routinely obtain pre-procedure IV access for almost all interventional procedures, others obtain IV access infrequently. Without compelling evidence to support either practice pattern, organizations and medical societies have been unable to provide clear recommendations. This survey study sought to describe (1) the current practice patterns concerning pre-procedure IV access utilization from a wide variety of interventional pain providers, and (2) practice patterns related to the use of an IV in the occurrence of emergency medical situations during interventional pain procedures.

## 2 Methods

Our local Institutional Review Board deemed this research to be exempt of need for approval as a survey study of physicians. A standardized survey was distributed via email to physician members of the Spine Intervention Society (SIS) on August 13, 2020 and remained open until October 13, 2020. Data was collected through administration of an anonymous survey constructed using Research Electronic Data Capture (REDCap). Respondents provided information regarding demographics and current practice patterns. Physicians who received the survey included current trainees, members of the American Boards of Medical Specialties or the American Osteopathic Association within the USA or Canada, or those who hold similar status in their country of residence in the fields of Anesthesiology, Physical Medicine and Rehabilitation (PM&R), Radiology, Neurology, Orthopedic Surgery or Neurosurgery. All responses were anonymous, and no identifying information was collected from survey respondents. The survey used to collect information is shown in appendix A.

### 2.1 Statistical analyses

Descriptive statistics were calculated as summary measures of the survey data. Specifically, frequency and percentage were used for categorical variables, while continuous variables were summarized using mean and standard deviation (SD). Monthly procedure volume and the number of yearly IV uses by demographics and training/clinical settings of physicians were examined, using the Wilcoxon-Mann-Whitney rank-sum tests (if grouping variable only had two categories) or the Kruskal-Wallis tests (if grouping variable had ​> ​two categories) [7,8]. In case of a statistically significant finding from the Kruskal-Wallis test, Dunn's test of multiple comparisons was conducted to further identify the significant between-group differences [[9], [10]]. The analysis also included the examination of the distributions of routine IV access when performing a procedure without sedation by physicians. Further, a binomial test was performed to examine the associations of practice setting to routine IV access for interventional procedures if they were practicing in a setting different from their current procedure location. Correlations between IV use and the number of monthly procedures performed were quantified with the Kendall's rank correlation coefficient or tau (*τ*). The number of physicians who had ever used IV to manage complications by procedure type was calculated for each anatomic location (cervical, thoracic, lumbar, and other). A complication rate was calculated for each procedure from the number of complications divided by the number of procedures in a typical month. In addition, complications rates were compared among the four anatomic locations and within each anatomic location, using two-sample tests of proportions with the Bonferroni correction. All the analyses were performed with Stata/MP 17.0 for Windows (StataCorp LLC, College Station, TX), and an α level of 0.05 was used for statistical significance.

## 3 Results

### 3.1 Demographics

The email containing the link to the study survey was sent to 7289 email addresses of current or expired members of the SIS. It is unknown how many duplicate email addresses were included (individuals with multiple email addresses on this list serve). Of those, 162 individuals started the survey and 141 of them completed it. Five individuals did not provide the answer on the number of procedures they performed in a given month for cervical, thoracic, lumbar, or other. After excluding these individuals, the 136 completed surveys were included for analysis. The demographic information of survey participants is listed in Table 1. Survey respondents were most commonly trained in PM&R (45.6%) or Anesthesiology (39.7%), fellowship-trained (77.9%), and practicing in a small group private practice (33.1%) or academic setting (26.5%), with 18.2 ​± ​11.5 years in practice. 47.1% of survey respondents indicated that they primary perform procedures in a CBPS followed by HBSC (25.7%) and ASC (24.3%).

**Table 1:** Demographic information of survey participants (*n* ​= ​136).

Characteristic	Frequency (%)
Practice Location	
Northeast	14 (10.3)
South	34 (25.0)
Midwest	20 (14.7)
West	37 (27.2)
Outside US	31 (22.8)
Primary Specialty	
Anesthesiology	54 (39.7)
PM&R	62 (45.6)
Radiology	6 (4.4)
Other	14 (10.3)
Fellowship trained	
Yes	106 (77.9)
No	30 (22.1)
Type of fellowship	
ACGME-accredited pain	44 (32.3)
ACGME-accredited sports	3 (2.2)
Non-ACGME accredited pain	15 (11.0)
Non-ACGME accredited spine/sports fellowship	19 (14.0)
Other fellowship	25 (18.4)
Missing	30 (22.1)
Primary practice setting	
Academic	36 (26.5)
Large group/hospital based	23 (16.9)
Government sponsored	3 (2.2)
Community clinic	2 (1.5)
Small group private practice	45 (33.1)
Solo private practice	21 (15.4)
Other	6 (4.4)
Primary procedure location for spinal interventions	
Clinic-based procedure suite	64 (47.1)
Hospital-based procedure suite	35 (25.7)
Ambulatory surgical center	33 (24.3)
Other	4 (2.9)
Years in practice [mean (SD)]	18.2 (11.5)

### 3.2 Monthly procedure volume

The survey respondents reported that they performed 111.4 ​± ​62.8 procedures on average per month. Lumbar spine procedures were most commonly performed (56.5 ​± ​30.1 procedures/month), followed by cervical spine procedures (23.1 ​± ​20.3 procedures/month), “other procedures” (26.3 ​± ​23.6 procedures/month) including large joint injections, bursa injections, peripheral nerve blocks, etc., and lastly thoracic procedures (5.5 ​± ​8.6 procedures/month). Of the respondents who had completed fellowship training (n ​= ​106), those who had completed a non-ACGME-accredited pain fellowship reported the highest volume of procedures per month (136 ​± ​63) followed by those who completed an ACGME-accredited pain fellowship (117 ​± ​68), though there was no significant association between type of fellowship and the number of total procedures per month (p ​= ​0.107) (Table 2). Further, no significant difference was found in the number of total procedures per month between the group of physicians who completed fellowship training and those who did not (p ​= ​0.435).

**Table 2:** Average number of total procedures per month by training and clinical setting.

Training and clinical setting	Average number of total procedures/month [mean (SD)]	*p*^a^
Primary specialty		0.105
Anesthesiology (n ​= ​54)	126 (67)	
PM&R (n ​= ​62)	105 (54)	
Radiology (n ​= ​6)	86 (47)	
Other (n ​= ​14)	96 (81)	
Type of fellowship		0.107
ACGME-accredited pain fellowship (n ​= ​44)	117 (68)	
ACGME-accredited sports fellowship (n ​= ​3)	89 (57)	
Non-ACGME-accredited pain fellowship (n ​= ​15)	136 (63)	
Non-ACGME Spine/Sports Fellowship (n ​= ​19)	105 (54)	
Other fellowship (n ​= ​25)	89 (68)	
Practice location		0.578
Northeast (n ​= ​14)	108 (49)	
South (n ​= ​34)	123 (71)	
Midwest (n ​= ​20)	122 (68)	
West (n ​= ​37)	108 (61)	
Outside US (n ​= ​31)	97 (58)	
Primary practice setting		0.043
Academic (n ​= ​36)	94 (57)	
Large Group/Hospital Based (n ​= ​23)	98 (51)	
Government-Sponsored (n ​= ​3)	121 (66)	
Community clinic (n ​= ​2)	128 (32)	
Small group private practice (n ​= ​45)	139 (72)	
Solo private practice (n ​= ​21)	99 (52)	
Other (n ​= ​6)	93 (54)	
Intervention setting		0.035
Clinic-based procedure suite (n ​= ​64)	117 (65)	
Hospital-based surgical center (n ​= ​35)	92 (64)	
Ambulatory surgical center (n ​= ​33)	125 (55)	
Other (n ​= ​4)	84 (47)	

Note: Values are mean (SD).

a From Kruskal-Wallis test.

Average numbers of total procedures per month differed by training and clinical setting (Table 2). Specifically, there was a significant difference in the numbers of total monthly procedures among the primary practice settings (*p* ​= ​0.043). According to the multiple comparisons with Dunn's tests, the number of total monthly procedures was significantly higher in small group private practice settings than in academic settings (139 ​± ​72 procedures/month vs. 94 ​± ​57 procedures/month; *p* ​= ​0.014). There were no significant differences in other pairwise comparisons of the primary practice setting (*p* ​> ​0.05). A significant difference in the numbers of total monthly procedures was also found among the intervention settings (*p* ​= ​0.035). Multiple comparisons with Dunn's tests revealed that the number of total monthly procedures was significantly higher in ASCs than in HBSCs (125 ​± ​55 procedures/month vs. 92 ​± ​64 procedures/month; *p* ​= ​0.020). There were no significant differences in other pairwise comparisons of the intervention settings (*p* ​> ​0.05).

### 3.3 Routine IV access

A bimodal distribution was noted for most procedures, with the majority of interventionalists obtaining routine IV access either 0–20% of the time or 81–100% of the time (Table 3). Routine IV access was obtained more frequently when performing cervical spine procedures, lumbar sympathetic blocks (LSB), spinal cord stimulator (SCS) trials, and splanchnic or hypogastric plexus blocks. Excluding cases where IVs were used for administering sedation agents, the only procedures which interventionalists obtained routine IV access more often than not were SCS trials and splanchnic or hypogastric plexus blocks. There was no significant difference between the likelihood of an IV being obtained when performing any given procedure and the following: geographical location, primary specialty, or type of fellowship (*p* ​> ​0.05).

**Table 3:**
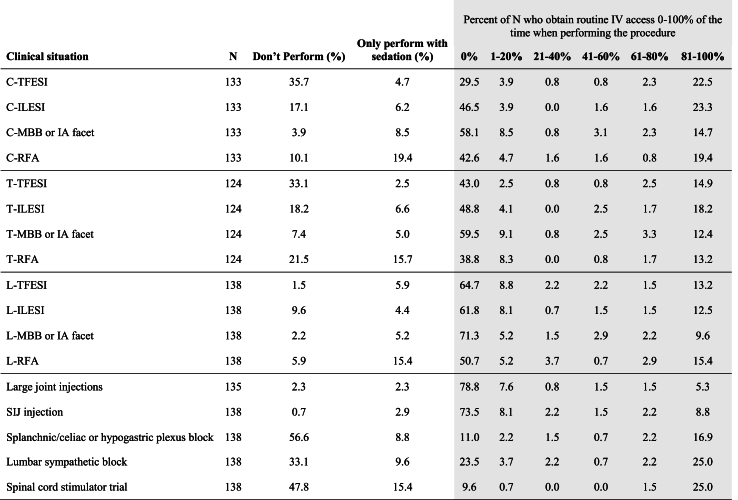
Percentage of the time routine IV access is obtained when performing procedure without sedation by survey respondents.

**Legend:** N ​= ​number of survey responders who answered the survey question; n ​= ​number of survey responders who answered the survey question and perform the procedure without sedation. C-TFESI ​= ​cervical transforaminal epidural steroid injection; C-ILESI ​= ​cervical interlaminar epidural steroid injection; C-MBB or IA facet ​= ​cervical medial branch block or cervical intraarticular facet injection; C-RFA ​= ​cervical radiofrequency ablation; T-TFESI ​= ​thoracic transforaminal epidural steroid injection; T-ILESI ​= ​thoracic interlaminar epidural steroid injection; T-MBB or IA facet ​= ​thoracic medial branch block or thoracic intraarticular facet injection; T-RFA ​= ​thoracic radiofrequency ablation; L-TFESI ​= ​lumbar transforaminal epidural steroid injection; L-ILESI ​= ​lumbar interlaminar epidural steroid injection; L-MBB or IA facet ​= ​lumbar medial branch block or lumbar intraarticular facet injection; L-RFA ​= ​lumbar radiofrequency ablation; SIJ injection ​= ​sacroiliac joint injection.

### 3.4 Practice setting

Respondents most commonly performed spinal interventions in a CBPS 47.1%), compared to HBSCs (25.7%) or ASCs (24.3%) (Table 1). Overall, most respondents reported they would obtain IV access the same amount regardless of practice setting (CBPS ​= ​32.4%, HBSC ​= ​47.8%, ASC ​= ​47.8.%). When data was organized into dichotomous variables (“less often” and “more often”) and “the same amount,” was excluded, trends emerged. A significantly higher proportion of respondents indicated that they would obtain IV access “more often” (*n* ​= ​34) if they were in a HBSC rather than “less often” (*n* ​= ​2; *p* ​< ​0.001). Similarly, a significantly higher proportion of respondents indicated that they would obtain IV access “more often” in an ASC (*n* ​= ​29) than “less often” (*n* ​= ​9; *p* ​< ​0.001). Contrary to this, a significantly higher proportion of respondents indicated that they would obtain routine IV access “less often” if they were in a CBPS (*n* ​= ​25) rather than “more often” (*n* ​= ​3; *p* ​< ​0.001) (Table 4).

**Table 4:** Proportion of respondents who said they would increase or decrease use of routine IV access for interventional procedures if they were practicing in a setting different from their current procedure location.

If you were to perform procedures in a clinic-based procedure suite, rather than your typical location, how often would you obtain IV access?			
Choice	f	%	*p*^a^
Less often	25	89.3	<0.001
More often	3	10.7	
**Total**	**31**	**100.0**	

If you were to perform procedures in a **hospital-based surgical center**, rather than your typical location, how often would you obtain IV access?			
Choice	f	%	*p*^a^
Less often	2	5.6	<0.001
More often	34	94.4	
**Total**	**36**	**100.0**	

If you were to perform procedures in an **ambulatory surgical center,** rather than your typical location, how often would you obtain IV access?			
Choice	f	%	*p*^a^
Less often	9	23.7	<0.001
More often	29	76.3	
**Total**	**38**	**100.0**	

a From binomial test.

### 3.5 Routine IV access by procedure volume

To determine whether providers who performed procedures more frequently were less likely to use IV's, the Kendall rank correlation coefficients (*τ*) were calculated. Those who reported performing a higher monthly volume of cervical procedures compared to other survey respondents were slightly less likely to obtain a routine IV for cervical interlaminar epidural steroid injection (ILESI) and cervical radiofrequency ablation (RFA) procedures (*τ* ​= ​−0.237 and −0.155 respectively; *p* ​< ​0.05) (Table 5). Physicians who perform thoracic level procedures were found to have no strong predilection for routine IV use when performing a thoracic level procedure (*p* ​> ​0.05). Physicians who perform a higher monthly volume of lumbar procedures compared to other survey respondents were found to place slightly fewer IVs for lumbar RFA procedures (*τ* ​= ​−0.149; *p* ​< ​0.05).

**Table 5:** Correlations of IV use and number of procedures performed monthly.

	Injections		
	Cervical	Thoracic	Lumbar
TFESI	−0.076	0.090	−0.023
ILESI	−0.237∗	−0.051	−0.097
MBB/IA facet	−0.110	0.082	−0.002
RFA	−0.155∗	−0.044	−0.149∗

Note: Values are Kendall's rank correlation coefficient or tau (*τ*).

∗*p* ​< ​0.05.

**Legend:** TFESI ​= ​transforaminal epidural steroid injection; ILESI ​= ​interlaminar epidural steroid injection; MBB/IA facet ​= ​medial branch block/intraarticular facet injection; RFA ​= ​radiofrequency ablation.

### 3.6 IV use during emergent medical situations

**Fig. 1:**
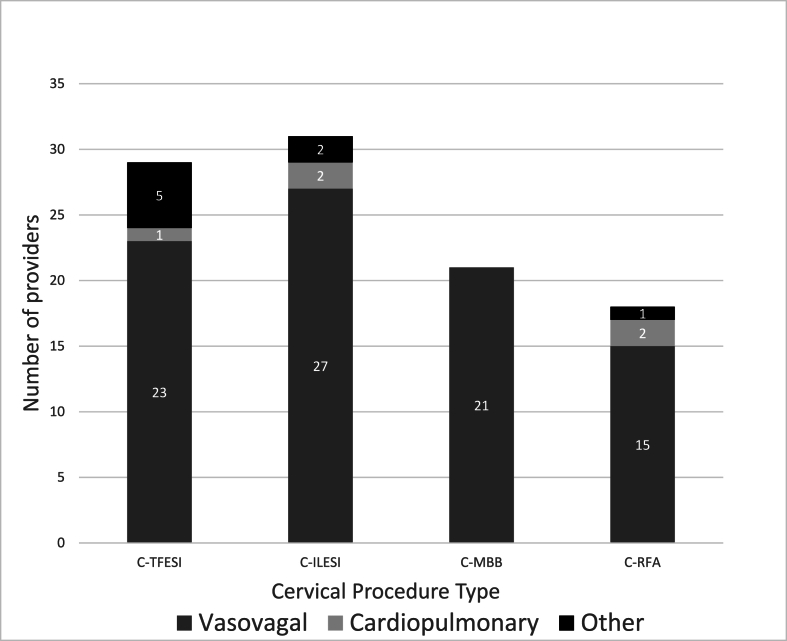
Number of providers who have ever used IV to manage cervical procedure complications by procedure type. Legend: C-TFESI= Cervical transforaminal epidural steroid injection; C-ILESI=Cervical interlaminar epidural steroid injection; C-MBB=Cervical medial branch block/intraarticular facet injection; C-RFA= Cervical radiofrequency ablation. *n* ​= ​99.

**Fig. 2:**
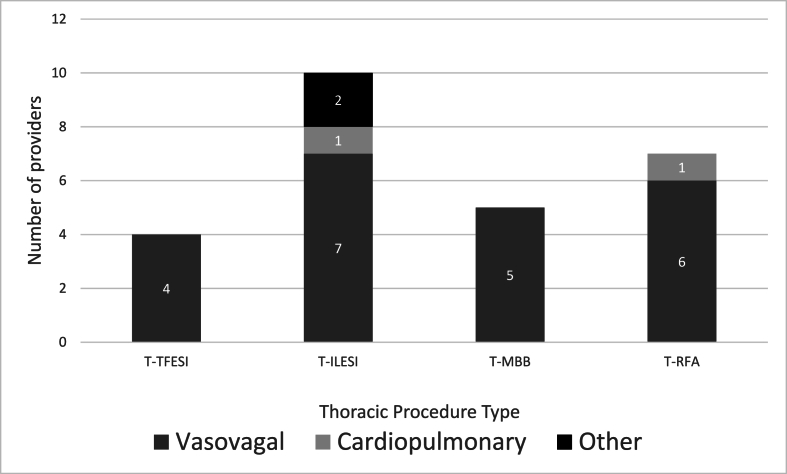
Number of providers who have ever used IV to manage thoracic procedure complications by procedure type. Legend: T-TFESI ​= ​Thoracic transforaminal epidural steroid injection; T-ILESI= Thoracic interlaminar epidural steroid injection; T-MBB ​= ​Thoracic medial branch block/intraarticular facet injection; T-RFA ​= ​Thoracic radiofrequency ablation. *n* ​= ​26.

**Fig. 3:**
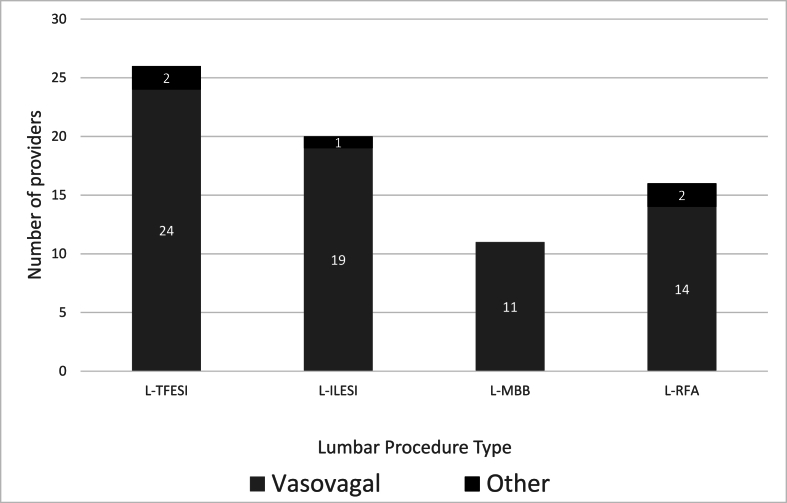
Number of providers who have ever used IV to manage lumbar procedure complications by procedure type. Legend: L-TFESI ​= ​Lumbar transforaminal epidural steroid injection; L-ILESI= Lumbar interlaminar epidural steroid injection; L-MBB ​= ​Lumbar medial branch block/intraarticular facet injection; L-RFA ​= ​Lumbar radiofrequency ablation. *n* ​= ​73. Note: there were no reported cardiopulmonary events requiring IV access for lumbar procedures in this survey.

**Fig. 4:**
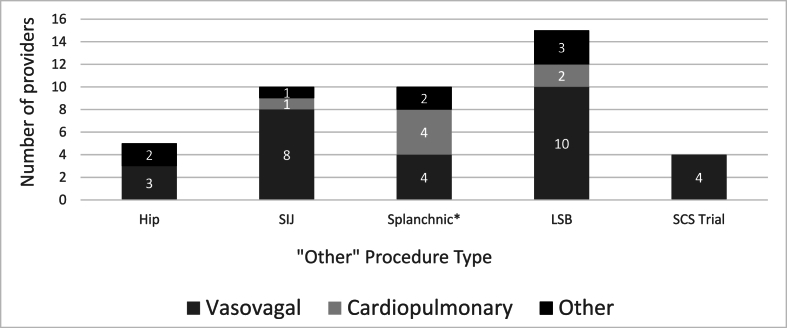
Number of providers who have ever used IV to manage other procedures complications by procedure type. Legend: SIJ=Sacral iliac joint; Splanchnic∗ ​= ​Splanchnic/celiac or hypogastric plexus block; LSB ​= ​lumbar sympathetic block; SCS ​= ​spinal cord stimulation trial. *n* ​= ​44.

Over half, 52.5% (*n* ​= ​74), of respondents reported using an IV during an emergency medical situation in their career. Of those, 48.6% (*n* ​= ​36) reported using an IV for a procedural complication within the past year. Primary specialty, fellowship location, practice setting, and intervention setting were not significantly associated with the number of emergent IVs utilized per year (*p* ​> ​0.05) (Table 6). The most common complication requiring IV use was a vasovagal event for all procedures except splanchnic/celiac or hypogastric plexus block in which an equal number of interventionalists used IVs for both vasovagal and cardiopulmonary events (Fig. 1, Fig. 2, Fig. 3, Fig. 4). Lumbar sympathetic blocks were more commonly associated with vasovagal events compared to cardiopulmonary or “other” events (Fig. 4). Utilization of IVs for emergent situations was most commonly reported in association with cervical spine procedures, lumbosacral transforaminal epidural steroid injection (TFESI), and lumbosacral ILESI. IV use for cardiopulmonary resuscitation was reported with splanchnic/celiac or hypogastric plexus block (*n* ​= ​4), LSB (*n* ​= ​2), cervical RFA (*n* ​= ​2), cervical ILESI (*n* ​= ​2), cervical TFESI (*n* ​= ​1), sacroiliac joint (SIJ) injection (*n* ​= ​1), thoracic RFA (*n* ​= ​1), and thoracic ILESI T1/2-T12/L1 (*n* ​= ​1). IV use for cardiopulmonary resuscitation was not reported for any lumbar spine procedures (Fig. 3). Use for other emergent management (i.e. seizure) was reported with cervical TFESI (*n* ​= ​5), LSB (*n* ​= ​3), splanchnic/celiac or hypogastric plexus block (*n* ​= ​2), intra-articular hip/glenohumeral joint injection (*n* ​= ​2), lumbosacral RFA (*n* ​= ​2), lumbosacral TFESI (*n* ​= ​2), thoracic ILESI (T1/2-T12/L1) (*n* ​= ​2), cervical ILESI (*n* ​= ​2), cervical RFA (*n* ​= ​1), lumbosacral ILESI (*n* ​= ​1), and SIJ injection (*n* ​= ​1) (Fig. 1, Fig. 2, Fig. 3, Fig. 4).

**Table 6:** IV use in emergency situations.

Training and clinical setting	Number of IV used per year	*p*^a^
Primary specialty		
Anesthesiology (n ​= ​37)	2.1 (4.0)	0.218
PM&R (n ​= ​26)	0.5 (0.7)	
Radiology (n ​= ​3)	0.3 (0.6)	
Other (neurology, neurosurgery, orthopedics, family medicine, other) (n ​= ​8)	0.9 (0.8)	
Location of fellowship		
Northeast (n ​= ​7)	0.7 (1.3)	0.340
South (n ​= ​15)	1.8 (3.9)	
Midwest (n ​= ​8)	0.3 (0.5)	
West (n ​= ​18)	0.9 (1.3)	
Outside US (n ​= ​26)	1.8 (3.8)	
Primary practice setting		
Academic (n ​= ​23)	1.3 (3.9)	0.430
Large Group/Hospital Based (n ​= ​11)	2.3 (4.5)	
Government-Sponsored (n ​= ​3)	1.0 (1.0)	
Community clinic (n ​= ​2)	0.5 (0.7)	
Small group private practice (n ​= ​20)	1.5 (1.5)	
Solo private practice (n ​= ​12)	0.4 (0.8)	
Other (n ​= ​3)	1.7 (2.9)	
Intervention setting		
Clinic-based procedure suite (n ​= ​31)	0.6 (0.9)	0.418
Hospital-based surgical center (n ​= ​23)	2.1 (4.8)	
Ambulatory surgical center (n ​= ​18)	1.6 (2.0)	
Other (n ​= ​2)	1.0 (1.4)	

a From Kruskal-Wallis test.

To better assess the relative risk of reported complications in which an IV was used by anatomic location (cervical, thoracic, lumbar, or other), we determined the number of interventionalists who reported a complication for a given procedural level and type and adjusted for the number of procedures performed in that area. After the Bonferroni correction, it was found that thoracic (24 out of 749 or 3.20%) and cervical (95 out of 3141 or 3.02%) procedures were associated with a significantly higher rate of use of an IV to manage a complication when compared to lumbar procedures (71 out of 7683 or 0.92%) or “other” procedures which included splanchnic/celiac or hypogastric plexus block, LSB, SCS, SIJ injections, and peripheral joints (41 out of 3574 or 1.15%, *p* ​< ​0.001) (Table 7, Table 8).

**Table 7:** Estimated complication rate by procedure.

Procedure	Number of complications (all providers)	Number of spine procedures in typical month (all providers)	Complication rate (%)
C-TFESI	28	3141	0.89
C-ILESI	29		0.92
C-MBB or IA Facet	21		0.67
C-RFA/Denervation	17		0.54
T-TFESI	4	749	0.53
T-ILESI	8		1.07
T-MBB or IA Facet	5		0.67
Thoracic RFA/Denervation	7		0.93
L-TFESI	25	7683	0.33
L- ILESI	19		0.25
L-MBB or IA Facet	11		0.14
L-RFA/Denervation	16		0.21
Intra-articular Hip/Glenohumeral Joint	4	3574	0.11
SIJ	9		0.25
Splanchnic/Celiac or Hypogastric Plexus Block	10		0.28
LSB	14		0.39
SCS	4		0.11

**Legend:** N. C-TFESI ​= ​cervical transforaminal epidural steroid injection; C-ILESI ​= ​cervical interlaminar epidural steroid injection; C-MBB or IA facet ​= ​cervical medial branch block or cervical intraarticular facet injection; C-RFA ​= ​cervical radiofrequency ablation; T-TFESI ​= ​thoracic transforaminal epidural steroid injection; T-ILESI ​= ​thoracic interlaminar epidural steroid injection; T-MBB or IA facet ​= ​thoracic medial branch block or thoracic intraarticular facet injection; T-RFA ​= ​thoracic radiofrequency ablation; L-TFESI ​= ​lumbar transforaminal epidural steroid injection; L-ILESI ​= ​lumbar interlaminar epidural steroid injection; L-MBB or IA facet ​= ​lumbar medial branch block or lumbar intraarticular facet injection; L-RFA ​= ​lumbar radiofrequency ablation; SIJ injection ​= ​sacroiliac joint injection; LSB ​= ​lumbar stellate block; SCS ​= ​spinal cord stimulator trial.

**Table 8:** Complication rate by anatomical spine region.

Location	Number of complications (all providers)	Number of spine procedures in typical month (all providers)	Complication rate (%)
Cervical	95	3141	3.02∗
Thoracic	24	749	3.20∗∗
Lumbar	71	7683	0.92
Other	41	3574	1.15

∗Significantly higher than lumbar or other (p ​< ​0.001) after Bonferroni correction.

∗∗Significantly higher than lumbar or other (p ​< ​0.001) after Bonferroni correction.

**Legend:** Cervical ​= ​cervical transforaminal epidural steroid injection, cervical interlaminar epidural steroid injection, cervical medial branch block or intraarticular facet injection, and cervical radiofrequency ablation/denervation; Thoracic ​= ​thoracic transforaminal epidural steroid injection, thoracic interlaminar epidural steroid injection, thoracic medial branch block or intraarticular facet injection, and thoracic radiofrequency ablation/denervation; Lumbar ​= ​lumbar transforaminal epidural steroid injection, lumbar interlaminar epidural steroid injection, lumbar medial branch block or intraarticular facet injection, and lumbar radiofrequency ablation/denervation; Other ​= ​intraarticular hip injection, glenohumeral joint injection, sacroiliac joint injection, splanchnic/celiac or hypogastric plexus block, lumbar sympathetic block, and spinal cord stimulator trial.

Within anatomical spine regions, cervical epidurals (transforaminal and interlaminar combined) were associated with a significantly higher estimated complication rate in which an IV was used than the combination of cervical facet procedures, including medial branch block (MBB), facet injection, and RFA (1.81% vs. 1.21%, *p* ​= ​0.049), though the difference was small (Table 9). Lumbar-epidurals (transforaminal and interlaminar combined) had a significantly higher complication rate than combination of lumbar MBB, facet injections, and RFA (0.57% vs. 0.35%; p ​= ​0.043). The estimated complication rates for thoracic and thoracic ILESI were identical at 1.60% (p ​= ​0.999). Within the ‘other’ group, LSB was associated with the highest rate of complications in which IV use was reported (0.39%), followed by splanchnic/celiac or hypogastric block (0.28%), SIJ injections (0.25%), and SCS trials (0.11%) and intra-articular hip/shoulder injections (0.11%) (Table 7).

**Table 9:** Complication rates of epidural steroid injections vs medial branch blocks/facet injections/RFA by anatomical location.

Procedures	Number of complications	Number of spine procedures in typical month	Complication rate (%)	*p*
C-TFESI ​+ ​C-ILESI	57	3141	1.81	0.049∗
C-MBB or IA Facet ​+ ​C-RFA/Denervation	38		1.21	
T-TFESI ​+ ​T- ILESI	12	749	1.60	0.999
T-MBB or IA Facet ​+ ​T-RFA/Denervation	12		1.60	
L-TFESI ​+ ​L-ILESI	44	7683	0.57	0.043∗
L-MBB or IA Facet ​+ ​L-RFA/Denervation	27		0.35	

∗Significant difference based on two-sample test of proportions.

**Legend:** C-TFESI ​+ ​C-ILESI ​= ​cervical transforaminal epidural steroid injection ​+ ​cervical interlaminar epidural steroid injection; C-MBB or IA Facet ​+ ​C- RFA/Denervation ​= ​cervical medial branch block or intraarticular facet injection ​+ ​cervical radiofrequency ablation/denervation; T-TFESI ​+ ​T- ILESI ​= ​thoracic transforaminal epidural steroid injection ​+ ​thoracic interlaminar epidural steroid injection; T-MBB or IA Facet ​+ ​T-RFA/Denervation ​= ​thoracic medial branch block or intraarticular facet injection ​+ ​thoracic radiofrequency ablation/denervation; L-TFESI ​+ ​L-ILESI ​= ​lumbar transforaminal epidural steroid injection ​+ ​lumbar interlaminar epidural steroid injection; L-MBB or IA Facet ​+ ​L- RFA/Denervation ​= ​lumbar medial branch block or intraarticular facet injection ​+ ​lumbar radiofrequency ablation/denervation.

## 4 Discussion

This survey study demonstrates wide variation amongst interventionalists for establishing IV access, with most providers obtaining routine IV access either 0–20% of the time or 81–100% of the time. This indicates that there is no clear consensus on when to place an IV for interventional procedures.

Overall, most interventionalists reported they did not routinely establish IV access for the majority of interventional pain procedures. Routine IV access was more commonly obtained for cervical spine procedures, LSB, splanchnic/celiac or hypogastric blocks, and SCS trials. This is likely due to the increased complexity of these procedures and the potential for more severe complications.

A large proportion of interventionalists disclosed that they do not routinely place IVs for cervical TFESIs or cervical ILESIs. This is surprising given the known potential risks of complications such as high spinal block in either cervical TFESI or ILESI and seizure from a cervical TFESI that includes local anesthetic [[11], [12], [13], [14]].

It is unclear why some interventionalists reported routine placement of IVs for large joint injections, lumbosacral MBBs, and SIJ injections. We speculate that these IVs are placed in patients who have a known history of vasovagal response during injections. Alternatively, depending on the interventionalist's local resources, it may be customary and simpler for nursing protocol to include IV placement in all patients present for a procedure.

No statistically significant, meaningful relationships were identified between establishing routine IV access for interventional procedures and the following demographic data: geographical location, primary specialty, type of fellowship, primary practice setting or intervention setting. This suggests that geography, training, and current practice setting do not influence routine IV placement practices.

The present data do indicate that the setting of procedures (CBPS, HBSC, or ASC) influences an interventionalist's choice as to whether to routinely obtain IV access. A significantly higher proportion of physicians that typically perform spinal interventions at CBPSs report that they would obtain IV access more often if they were to perform procedures in an ASC (90%), compared to those typically performing interventions at HBSCs (14.3%). It is possible that those who perform procedures predominantly in a clinic setting may move more quickly through procedures than those at an ASC or HBSC and find that it is not an efficient use of their time or clinic staff time to routinely place IVs.

Interventionalists who reported performing a higher monthly volume of cervical procedures compared to other survey respondents were slightly less likely to routinely obtain IV access for cervical ILESI and cervical RFA procedures. Furthermore, those who performed a higher monthly volume of lumbar procedures compared to other survey respondents were found to place fewer IVs for lumbar RFA procedures. This could reflect a comfort and confidence level obtained with certain spine procedures secondary to volume repetition frequency. Alternatively, busier interventionalists might avoid routine IV access for some procedures to improve practice efficiency. If this is the case, we encourage interventionalists to carefully weigh the safety disadvantages of a lack of pre-procedural IV access with the perceived improvement in efficiency, particularly for cervical-level procedures and especially in patients with medical co-morbidities that increase their risk of a potential emergent medical event that would require management with urgent IV medications and/or fluids. Notably, IV use for cardiopulmonary arrest was reported in association with splanchnic/celiac or hypogastric plexus block, LSB, and cervical spine procedures including RFA and ILESI. IV use for other emergent situations was most common with cervical TFESI and LSB.

To estimate a procedure specific complication rate, the number of providers reported a complication for a given procedure level and type was adjusted for the number of monthly procedures performed in that anatomic area. Overall, calculated complication rates requiring use of an IV for interventional spine procedures were low. Based on anatomical spine regions, lumbar spine had the lowest estimated complication rate rather required IV use (1.09%), followed by the cervical spine (2.93%), and the thoracic spine (3.13%). The aggregate data indicates that thoracic procedures (*n* ​= ​766) are performed much less commonly than lumbar (*n* ​= ​7853) or cervical procedures (*n* ​= ​3241) in a typical month by survey responders. It is possible that the lower volume of these procedures performed by interventionists may factor into the higher complication rate observed, if an interventionists is less comfortable and experienced with cervical or thoracic level procedures.

Of note, it seemed curious that SCS trial and sympathetic plexus block complication rate in which an IV was used was exceedingly low, especially when compared to rates for lumbar procedures such as lumbar TFESI or lumbar RFA. We speculate that this is likely due to the fact that most patients undergoing SCS trials or sympathetic plexus block already have an IV in place for moderate sedation and/or administration of IV antibiotics or fluids in anticipation of the possibility of vasovagal reaction and/or hypotension, so emergent IV use is less common.

There are several limitations to this study. This survey was only available to members of SIS and practice patterns might differ outside of members of this society. Further, only 162 members opened the survey email and 141 members actually filled out the survey which may limit generalizability. Members of SIS who chose to complete the study may have a particular interest in this topic and thus might have responded differently than others. The data for this study was also collected during the SARS-CoV-2 global pandemic which may have impacted practice patterns. The calculated complications rates in which an IV was used might underestimate the true complication rates, as any provider who had multiple complications for the same procedure was only counted once. However, as the overall complication rate in interventional procedures as reported in this survey was very low (almost half of respondents reporting never using an IV in an emergent situation), the estimated complication rate still provides a useful observation and may inform the use of IV access in different procedures. Additionally, we did not differentiate in our question about use of IVs for emergencies as to whether an IV placed pre-procedure was used as opposed to the need for placement of an IV because of an emergency. Secondly, when we asked about the percentage of time providers obtain IV access when performing various procedures without sedation, we included 81–100% of the time as an option but not “always/100% of the time” as an option. Thus, we do not know how many individuals placed IVs by default, 100% of the time, for non-sedation procedures. Lastly, as with any survey study, data is based upon respondent recall and was not directly measured.

## 5 Conclusion

This survey study of SIS members demonstrates wide variation in practice patterns of physicians in establishing routine IV access prior to interventional pain procedures. Most interventionalists surveyed reported not routinely establishing IV access for the majority of interventional pain procedures, but IV access is more frequently obtained for cervical spine procedures, LSB, splanchnic/celiac or hypogastric blocks, and SCS trials. Procedural setting appears to influence physician decision-making regarding routine IV access, with physicians less likely to obtain routine IV access in clinic-based procedure suites than hospital-based surgery centers or ambulatory surgical centers. Complications prompting IV access are rare in interventional pain procedures, but are likely to happen over the course of a career.

## Funding

This research did not receive any specific grant from funding agencies in the public, commercial, or not-for-profit sectors.

## Declaration of competing interest

The authors declare that they have no known competing financial interests or personal relationships that could have appeared to influence the work reported in this paper.

## References

[1] Starr J.B., Gold L., McCormick Z., Suri P., Friedly J. (2019). Trends in lumbar radiofrequency ablation utilization from 2007 to 2016. Spine J.

[2] Beckworth W.J., Jiang M., Hemingway J., Hughes D., Staggs D. (2016). Facet injection trends in the Medicare population and the impact of bundling codes. Spine J.

[3] Friedly J., Chan L., Deyo R. (2008). Geographic variation in epidural steroid injection use in medicare patients. The Journal of Bone and Joint Surgery-American.

[4] McCormick Z.L., Popescu A., Smith C.C. (2017). Spine intervention society's patient safety committee. Fact finders for patient safety: routine intravenous access for epidural steroid injections without sedation. Pain Med.

[5] Fields J.M., Piela N.E., Ku B.S. (2014). Association between multiple IV attempts and perceived pain levels in the emergency department. J Vasc Access.

[6] Dychter S.S., Gold D.A., Carson D., Haller M. (2012). Intravenous therapy. J Infusion Nurs.

[7] Mann H.B., Whitney D.R. (1947). On a test of whether one of two random variables is stochastically larger than the other. Ann Math Stat.

[8] Wilcoxon F. (1945). Individual comparisons by ranking methods. Biometrics Bull.

[9] Kruskal W.H., Wallis W.A. (1952). Use of ranks in one-criterion variance analysis. J Am Stat Assoc.

[10] Dunn O.J. (1964). Multiple comparisons using rank sums. Technometrics.

[11] Malhotra G., Abbasi A., Rhee M. (2009). Complications of transforaminal cervical epidural steroid injections. Spine.

[12] Epstein N.E. (2018). Major risks and complications of cervical epidural steroid injections: an updated review. Surg Neurol Int.

[13] Epstein N. (2013). The risks of epidural and transforaminal steroid injections in the Spine: commentary and a comprehensive review of the literature. Surg Neurol Int.

[14] Abbasi A., Malhotra G., Malanga G., Elovic E.P., Kahn S. (2007). Complications of interlaminar cervical epidural steroid injections. Spine.

