# Supplementary material for: Is routine intravenous access commonly established before interventional pain procedures? Results of a spine intervention society practice pattern survey
Source: Interv Pain Med. 2023 Apr 5;2(2):100246. doi: 10.1016/j.inpm.2023.100246 (PMC11372991; doi:10.1016/j.inpm.2023.100246)
Supplement: Multimedia component 1 [file mmc1.pdf]

IV use study

Please complete the survey below.

Thank you!

Where do you predominantly practice?	◯ Northeast (PA, NJ, NY, CT, RI, MA, VT, NH, ME)<br>◯ South (TX, OK, AR, LA, MS, AL, TN, KY, FL, GA, SC, NC, VA, DC, MD, DE, WV)<br>◯ Midwest (ND, S,D, NE, KS, MN, IA, MO, WI, IL, IN,MI, OH)<br>◯ West (NM, AZ, CO, UT, NV, CA, WY, MT, ID, OR, WA, AK, HI)<br>◯ Outside US
What is your primary specialty?	◯ Anesthesiology<br>◯ PM&R<br>◯ Radiology<br>◯ Other (neurology, neurosurgery, orthopaedics, family medicine, other)
If you answered other please specify your primary practice in the text box provided.<br><br>ex: (neurology, neurosurgery, orthopaedics, family medicine, other)	__________________________________
Did you complete a Fellowship?	◯ Yes<br>◯ No
If you completed a fellowship please define what type of fellowship you completed from the following options:	◯ ACGME-accredited pain fellowship<br>◯ ACGME-accredited sports fellowship<br>◯ Non-ACGME-accredited pain fellowship<br>◯ Non-ACGME Spine/Sports Fellowship<br>◯ Other fellowship
If you answered other fellowship above please provide more details in text box below:	__________________________________

Practice Type

Since the completion of your medical training, how many years have you been in practice?

◯ Still in Training
◯ 1
◯ 2
◯ 3
◯ 4
◯ 5
◯ 6
◯ 7
◯ 8
◯ 9
◯ 10
◯ 11
◯ 12
◯ 13
◯ 14
◯ 15
◯ 16
◯ 17
◯ 18
◯ 19
◯ 20
◯ 21
◯ 22
◯ 23
◯ 24
◯ 25
◯ 26
◯ 27
◯ 28
◯ 29
◯ 30
◯ 31
◯ 32
◯ 33
◯ 34
◯ 35
◯ 36
◯ 37
◯ 38
◯ 39
◯ 40
◯ 41
◯ 42
◯ 43
◯ 44
◯ 45
◯ 46
◯ 47
◯ 48
◯ 49
◯ 50
◯ 51+

What is your primary practice setting?

◯ Academic
◯ Large Group/Hospital Based
◯ Government-Sponsored
◯ Community clinic
◯ Small group private practice
◯ Solo private practice
◯ Other

In what setting do you typically perform spinal interventions	◯ Clinic-based procedure suite<br>◯ Hospital-based surgical center<br>◯ Ambulatory surgical center<br>◯ Other
If you answered other above please provide more details in the text box below	__________________________________
If you were to perform procedures in a clinic-based procedure suite, rather than your typical location, how often would you obtain IV access?	◯ Significantly less often<br>◯ Slightly less often<br>◯ The same amount<br>◯ Slightly more often<br>◯ Significantly more often
If you were to perform procedures in a hospital-based surgical center, rather than your typical location, how often would you obtain IV access?	◯ Significantly less often<br>◯ Slightly less often<br>◯ The same amount<br>◯ Slightly more often<br>◯ Significantly more often
If you were to perform procedures in a ambulatory surgical center, rather than your typical location, how often would you obtain IV access?	◯ Significantly less often<br>◯ Slightly less often<br>◯ The same amount<br>◯ Slightly more often<br>◯ Significantly more often

Procedures Performed

For all of the following questions - If you offer this procedure but perform it less than once per month, please select 1.

How many Cervical Spine Procedures do you perform in a typical month?

◯ 0 ◯ 1 ◯ 2 ◯ 3
◯ 4 ◯ 5 ◯ 6 ◯ 7
◯ 8 ◯ 9 ◯ 10 ◯ 11
◯ 12 ◯ 13 ◯ 14 ◯ 15
◯ 16 ◯ 17 ◯ 18 ◯ 19
◯ 20 ◯ 21 ◯ 22 ◯ 23
◯ 24 ◯ 25 ◯ 26 ◯ 27
◯ 28 ◯ 29 ◯ 30 ◯ 31
◯ 32 ◯ 33 ◯ 34 ◯ 35
◯ 36 ◯ 37 ◯ 38 ◯ 39
◯ 40 ◯ 41 ◯ 42 ◯ 43
◯ 44 ◯ 45 ◯ 46 ◯ 47
◯ 48 ◯ 49 ◯ 50 ◯ 51
◯ 52 ◯ 53 ◯ 54 ◯ 55
◯ 56 ◯ 57 ◯ 58 ◯ 59
◯ 60 ◯ 61 ◯ 62 ◯ 63
◯ 64 ◯ 65 ◯ 66 ◯ 67
◯ 68 ◯ 69 ◯ 70 ◯ 71
◯ 72 ◯ 73 ◯ 74 ◯ 75
◯ 76 ◯ 77 ◯ 78 ◯ 79
◯ 80 ◯ 81 ◯ 82 ◯ 83
◯ 84 ◯ 85 ◯ 86 ◯ 87
◯ 88 ◯ 89 ◯ 90 ◯ 91
◯ 92 ◯ 93 ◯ 94 ◯ 95
◯ 96 ◯ 97 ◯ 98 ◯ 99
◯ 100+

How many Thoracic Spine Procedures do you perform in a typical month?

◯ 0 ◯ 1 ◯ 2 ◯ 3
◯ 4 ◯ 5 ◯ 6 ◯ 7
◯ 8 ◯ 9 ◯ 10 ◯ 11
◯ 12 ◯ 13 ◯ 14 ◯ 15
◯ 16 ◯ 17 ◯ 18 ◯ 19
◯ 20 ◯ 21 ◯ 22 ◯ 23
◯ 24 ◯ 25 ◯ 26 ◯ 27
◯ 28 ◯ 29 ◯ 30 ◯ 31
◯ 32 ◯ 33 ◯ 34 ◯ 35
◯ 36 ◯ 37 ◯ 38 ◯ 39
◯ 40 ◯ 41 ◯ 42 ◯ 43
◯ 44 ◯ 45 ◯ 46 ◯ 47
◯ 48 ◯ 49 ◯ 50 ◯ 51
◯ 52 ◯ 53 ◯ 54 ◯ 55
◯ 56 ◯ 57 ◯ 58 ◯ 59
◯ 60 ◯ 61 ◯ 62 ◯ 63
◯ 64 ◯ 65 ◯ 66 ◯ 67
◯ 68 ◯ 69 ◯ 70 ◯ 71
◯ 72 ◯ 73 ◯ 74 ◯ 75
◯ 76 ◯ 77 ◯ 78 ◯ 79
◯ 80 ◯ 81 ◯ 82 ◯ 83
◯ 84 ◯ 85 ◯ 86 ◯ 87
◯ 88 ◯ 89 ◯ 90 ◯ 91
◯ 92 ◯ 93 ◯ 94 ◯ 95
◯ 96 ◯ 97 ◯ 98 ◯ 99
◯ 100+

How many Lumbar or Sacral Spine Procedures do you perform in a typical month?

○ 0 ○ 1 ○ 2 ○ 3
○ 4 ○ 5 ○ 6 ○ 7
○ 8 ○ 9 ○ 10 ○ 11
○ 12 ○ 13 ○ 14 ○ 15
○ 16 ○ 17 ○ 18 ○ 19
○ 20 ○ 21 ○ 22 ○ 23
○ 24 ○ 25 ○ 26 ○ 27
○ 28 ○ 29 ○ 30 ○ 31
○ 32 ○ 33 ○ 34 ○ 35
○ 36 ○ 37 ○ 38 ○ 39
○ 40 ○ 41 ○ 42 ○ 43
○ 44 ○ 45 ○ 46 ○ 47
○ 48 ○ 49 ○ 50 ○ 51
○ 52 ○ 53 ○ 54 ○ 55
○ 56 ○ 57 ○ 58 ○ 59
○ 60 ○ 61 ○ 62 ○ 63
○ 64 ○ 65 ○ 66 ○ 67
○ 68 ○ 69 ○ 70 ○ 71
○ 72 ○ 73 ○ 74 ○ 75
○ 76 ○ 77 ○ 78 ○ 79
○ 80 ○ 81 ○ 82 ○ 83
○ 84 ○ 85 ○ 86 ○ 87
○ 88 ○ 89 ○ 90 ○ 91
○ 92 ○ 93 ○ 94 ○ 95
○ 96 ○ 97 ○ 98 ○ 99
○ 100+

How many Other Interventional Procedures (eg. large joint/bursa injection, peripheral nerve block, etc.) do you perform in a typical month?

○ 0 ○ 1 ○ 2 ○ 3
○ 4 ○ 5 ○ 6 ○ 7
○ 8 ○ 9 ○ 10 ○ 11
○ 12 ○ 13 ○ 14 ○ 15
○ 16 ○ 17 ○ 18 ○ 19
○ 20 ○ 21 ○ 22 ○ 23
○ 24 ○ 25 ○ 26 ○ 27
○ 28 ○ 29 ○ 30 ○ 31
○ 32 ○ 33 ○ 34 ○ 35
○ 36 ○ 37 ○ 38 ○ 39
○ 40 ○ 41 ○ 42 ○ 43
○ 44 ○ 45 ○ 46 ○ 47
○ 48 ○ 49 ○ 50 ○ 51
○ 52 ○ 53 ○ 54 ○ 55
○ 56 ○ 57 ○ 58 ○ 59
○ 60 ○ 61 ○ 62 ○ 63
○ 64 ○ 65 ○ 66 ○ 67
○ 68 ○ 69 ○ 70 ○ 71
○ 72 ○ 73 ○ 74 ○ 75
○ 76 ○ 77 ○ 78 ○ 79
○ 80 ○ 81 ○ 82 ○ 83
○ 84 ○ 85 ○ 86 ○ 87
○ 88 ○ 89 ○ 90 ○ 91
○ 92 ○ 93 ○ 94 ○ 95
○ 96 ○ 97 ○ 98 ○ 99
○ 100+

Total monthly procedures (calculated/hidden) ______________________________

When performing the following cervical procedures WITHOUT sedation, what percentage of the time do you typically obtain IV access?

[TF = Transforaminal Epidural Steroid Injection, IL = Interlaminar, ESI = Epidural steroid injection, IA = Intra-articular, MBB = Medial Branch Block, RFA = Radiofrequency ablation, Proc. = Procedure]

	Don't Perform Proc.	Only Perform with Sedation	0%	1-20%	21-40%	41-60%	61-80%	81-100%
Cervical TFESI (C3-8)	○	○	○	○	○	○	○	○
Cervical IL ESI	○	○	○	○	○	○	○	○
Cervical MBB or IA Facet Injection	○	○	○	○	○	○	○	○
Cervical RFA	○	○	○	○	○	○	○	○

When performing the following thoracic procedures WITHOUT sedation, what percentage of the time do you typically obtain IV access?

[TF = Transforaminal Epidural Steroid Injection, IL = Interlaminar, ESI = Epidural steroid injection, IA = Intra-articular, MBB = Medial Branch Block, RFA = Radiofrequency ablation, Proc. = Procedure]

	Don't Perform Proc.	Only Perform with Sedation	0%	1-20%	21-40%	41-60%	61-80%	81-100%
Thoracic TFESI (T1-T12)	◯	◯	◯	◯	◯	◯	◯	◯
Thoracic ILESI	◯	◯	◯	◯	◯	◯	◯	◯
Thoracic MBB or IA Facet Injection	◯	◯	◯	◯	◯	◯	◯	◯
Thoracic RFA	◯	◯	◯	◯	◯	◯	◯	◯

When performing the following lumbosacral procedures WITHOUT sedation, what percentage of the time do you typically obtain IV access?

[TF = Transforaminal Epidural Steroid Injection, IL = Interlaminar, ESI = Epidural steroid injection, IA = Intra-articular, MBB = Medial Branch Block, RFA = Radiofrequency ablation, Proc. = Procedure]

	Don't Perform Proc.	Only Perform with Sedation	0%	1-20%	21-40%	41-60%	61-80%	81-100%
Lumbosacral TFESI (L1-S1)	○	○	○	○	○	○	○	○
Lumbosacral IL ESI	○	○	○	○	○	○	○	○
Lumbosacral MBB or IA Facet Injection	○	○	○	○	○	○	○	○
Lumbosacral RFA	○	○	○	○	○	○	○	○

When performing the following procedures WITHOUT sedation, what percentage of the time do you typically obtain IV access?

[TF = Transforaminal Epidural Steroid Injection, IL = Interlaminar, ESI = Epidural steroid injection, IA = Intra-articular, MBB = Medial Branch Block, RFA = Radiofrequency ablation, Proc. = Procedure]

	Don't Perform Proc.	Only Perform with Sedation	0%	1-20%	21-40%	41-60%	61-80%	81-100%
Large Joint Injection	○	○	○	○	○	○	○	○
Sacroiliac Joint Injection	○	○	○	○	○	○	○	○
Splanchnic/Celiac or Hypogastric Plexus Block	○	○	○	○	○	○	○	○
Lumbar Sympathetic Block	○	○	○	○	○	○	○	○
Spinal Cord Stimulator Trial	○	○	○	○	○	○	○	○

IV use

Have you ever used an IV for something other than sedation (e.g. emergency resuscitation)?

- ◯ Yes
- ◯ No

Approximately how many times in the past year have you used an IV for a procedural complication?

- ◯ 0
- ◯ 1
- ◯ 2
- ◯ 3
- ◯ 4
- ◯ 5
- ◯ 6
- ◯ 7
- ◯ 8
- ◯ 9
- ◯ 10
- ◯ 11
- ◯ 12
- ◯ 13
- ◯ 14
- ◯ 15
- ◯ 16
- ◯ 17
- ◯ 18
- ◯ 19
- ◯ 20
- ◯ 21
- ◯ 22
- ◯ 23
- ◯ 24
- ◯ 25

Approximately how many times in your career have you used an IV for a procedural complication?

- ◯ 1
- ◯ 2
- ◯ 3
- ◯ 4
- ◯ 5
- ◯ 6
- ◯ 7
- ◯ 8
- ◯ 9
- ◯ 10
- ◯ 11
- ◯ 12
- ◯ 13
- ◯ 14
- ◯ 15
- ◯ 16
- ◯ 17
- ◯ 18
- ◯ 19
- ◯ 20
- ◯ 21
- ◯ 22
- ◯ 23
- ◯ 24
- ◯ 25

IVs used per year (calculated)

____

Please select any situation where you have used an IV for a procedural complication. Select all that apply (okay to leave blank if none apply).

	Yes - for a vasovagal event	Yes - for cardiopulmonary resuscitation	Yes - for other emergency management (eg. seizure)
Cervical TFESI (C3-C8)	☐	☐	☐
Cervical IL ESI	☐	☐	☐
Cervical MBB or IA Facet Injection	☐	☐	☐
Cervical RFA/Denervation	☐	☐	☐
Thoracic TFESI (T1-T12)	☐	☐	☐
Thoracic IL ESI (T1/2-T12/L1)	☐	☐	☐
Thoracic MBB or IA Facet Injection	☐	☐	☐
Thoracic RFA/Denervation	☐	☐	☐
Lumbosacral TFESI	☐	☐	☐
Lumbosacral IL ESI	☐	☐	☐
Lumbar MBB or IA Facet Injection	☐	☐	☐
Lumbosacral RFA/Denervation	☐	☐	☐
Intra-articular Hip/Glenohumeral Joint	☐	☐	☐
SI Joint Injection	☐	☐	☐
Splanchnic/Celiac or Hypogastric Plexus Block	☐	☐	☐
Lumbar Sympathetic Block	☐	☐	☐
SCS Trial	☐	☐	☐

If you selected "Yes - for other emergency management" in the previous question, please note what the procedural complication(s) was/were. __________________________________

If you have selected more than one of these, please describe the all complications you have encountered, separated by commas (eg. seizure, vomiting, etc.)
